# A Dual pH/O_2_ Sensing Film Based on Functionalized Electrospun Nanofibers for Real-Time Monitoring of Cellular Metabolism

**DOI:** 10.3390/molecules27051586

**Published:** 2022-02-28

**Authors:** Dongyan Zhou, Hongtian Liu, Juewei Ning, Ge Cao, He Zhang, Mengyu Deng, Yanqing Tian

**Affiliations:** Department of Materials Science and Engineering, Southern University of Science and Technology, No. 1088 Xueyuan Blvd., Xili, Nanshan District, Shenzhen 518055, China; 11930937@mail.sustech.edu.cn (D.Z.); lht737512159@163.com (H.L.); jwning@gia.cas.cn (J.N.); 11749286@mail.sustech.edu.cn (G.C.); 19B909123@stu.hit.edu.cn (H.Z.)

**Keywords:** dual sensor, oxygen, pH sensor, electrospinning, cellular metabolism

## Abstract

Real-time monitoring of dissolved oxygen (DO) and pH is of great significance for understanding cellular metabolism. Herein, a dual optical pH/O_2_ sensing membrane was prepared by the electrospinning method. Cellulose acetate (CA) and poly(ε-caprolactone) (PCL) nanofiber membrane blended with platinum (II)-5,10,15,20-tetrakis-(2,3,4,5,6-pentafluorophenyl)-porphyrin (PtTFPP) was used as the DO sensing matrix, upon which electrospun nanofiber membrane of chitosan (CS) coupled with fluorescein 5-isothiocyanate (FITC) was used as the pH sensing matrix. The electrospun sensing film prepared from biocompatible biomaterials presented good response to a wide range of DO concentrations and physiological pH. We used it to monitor the exracellular acidification and oxygen consumption levels of cells and bacteria. This sensing film can provide a luminescence signal change as the DO and pH change in the growth microenvironment. Due to its advantages of good biocompatibility and high stability, we believe that the dual functional film has a high value in the field of biotechnology research.

## 1. Introduction

The monitoring of metabolite parameters associated with cellular growth is essential for biological and clinical studies [1]. During the processes of cellular respiration and metabolism, the consumption of oxygen and the production of metabolites will lead to a change in the concentration of dissolved oxygen (DO) and pH level in the culture microenvironment [2,3,4,5]. Therefore, sensors for real-time monitoring of DO and pH in the cellular growth microenvironment are of great significance.

Optical sensors based on reversible luminescence changes in specific indicators show high potential for environmental detection, due to the advantages of fast, simple and easy-to-read signals [6]. These sensors composed with probes and polymer matrices can clearly reflect the environmental changes through luminescence variations. For the selection of biosensing matrix materials, biobased polymers from natural sources have inherent advantages, such as low cost, nontoxicity, biodegradability and eco-friendliness, which make them attractive in the field of biomonitoring. 

Cellulose acetate (CA), the most abundant biobased material, has attracted the interest of scientists as an oxygen-sensitive matrix due to its high stability, simple molding process and easy modification [7,8,9]. However, its sensitivity to oxygen is still inadequate, because of its poor oxygen permeability [10]. Some attempts have been made to address this issue. For example, polymethyl methacrylate [7] and SiO_2_ [11] were used to improve the response performance of CA [10], but both of them are nondegradable materials, which limited their application. Hence, searching for modified materials with biodegradability is of great significance to green chemical and biosensing. In addition, chitosan (CS) was chosen as a pH sensing matrix, which is also a natural material with biodegradability [12]. CS is an ideal material for sensor applications due to easy modification of its structure and physiological function diversity.

Extensive work has been done on smart optical dual-response sensor films for oxygen and pH detection [13,14,15]. The integration of dual optical sensors into thin films generally takes two kinds of arrangements, one is to incorporate all the probes into a single monolayer, and the other is to embed the probes into two different films, respectively [16]. Compared to single-layer sensing films, two-layer sensing films can achieve optimal separation of the luminescences generated by DO and pH sensing systems [17]. Thus, we use double-layer electrospun films to integrate DO and pH sensing functions. The use of electrospun films generates films with large surface–volume ratios for achieving high sensitivity [18].

In this study, biodegradable poly(ε-caprolactone) (PCL) was blended with CA as an oxygen-sensitive matrix for obtaining electrospun DO sensor films. The oxygen-sensitive probe platinum (II)-5,10,15,20-tetrakis-(2,3,4,5,6-pentafluorophenyl)-porphyrin (PtTFPP) is strongly hydrophobic and can be directly doped in the polymer mixture of hydrophobic CA and PCL. Furthermore, the pH sensing property was integrated into the membrane by electrospinning CS, which was functionalized with a pH probe of fluorescein 5-isothiocyanate (FITC). FITC is one of the most commonly used pH indicators, and can be grafted on CS by the reaction of amino groups of chitosan with the isothiocyanate group of FITC [19]. Both the chosen probes respond solely to their specific target to alleviate interferences from other species in the applied environmental conditions. All the materials selected are environmentally friendly and nontoxic.

The resulting dual-response composite nanofiber films have the potential for intelligent monitoring because of their good response to DO and pH. The functionalized films were also used to monitor cellular respiration and metabolism, demonstrating the real-time biomonitoring capability of the sensing films. 

## 2. Materials and Methods

### 2.1. Materials

CA (MW = 3 × 10^4^ Da), PCL (MW = 8 × 10^4^ Da) and CS (MW = 3 × 10^5^ Da, DD = 92%) were obtained from Macklin Co., Ltd. (Shanghai, China). PtTFPP was supplied by Frontier Scientific (Logan, UT, USA). FITC was obtained from Sigma-Aldrich Co. (Saint Luis, MO, USA). N,N’-dimethylformamide (DMF), dichloromethane (DCM) and all other chemicals were supplied by Sinopharm Chemical Reagent Co., Ltd. (Shanghai, China). 

### 2.2. Synthesis of CS-FITC

The CS-FITC conjugated solution was prepared by adding the FITC solution (2 mg/mL in ethanol) into the CS solution (10 mg/mL in 0.1 M acetic acid), then stirred for 24 h in the dark. After the reaction, unreacted FITC was removed in DI water by dialysis (cut off: 8000). Finally, the CS-FITC powder with a yield of 90% was obtained by lyophilization.

### 2.3. Preparation of Electrospun Nanofibrous Matrix

#### 2.3.1. Preparation of CA&PCL Electrospun Nanofibrous Matrix (F1) for Oxygen Sensing

**Scheme 1:**
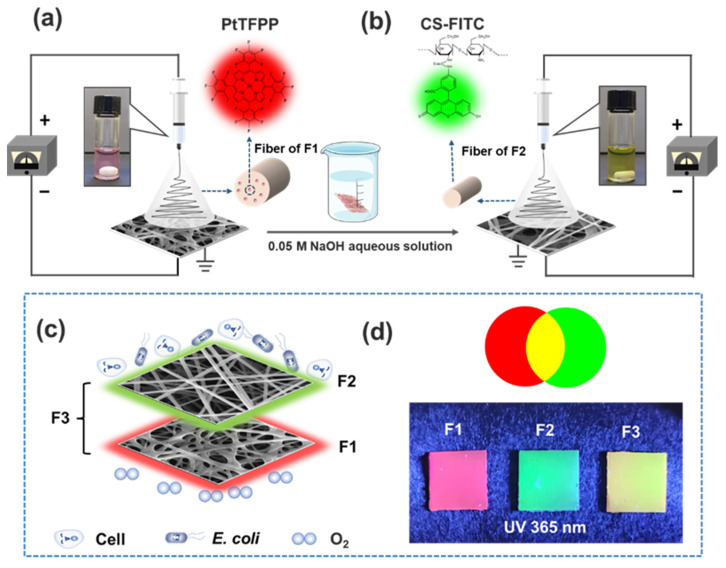
The fabrication of F1 (PCL&CA-PtTFPP, (**a**)), F2 (CS-FITC, (**b**)) and F3 (PCL&CA-PtTFPP/CS-FITC, (**c**)) nanofibrous membranes via electrospinning. The emission images of three films excited at UV 365 nm (**d**).

As shown in Table 1, five different sensing films (F1–F5) with variable compositions were prepared by electrospinning. CA (10 wt%) solution was prepared by dissolving CA in DMF. PCL (8 wt%) solution was prepared by dissolving PCL in a mixture of DCM and DMF with a volume ratio of 2:1. Mixing CA and PCL solutions yielded a mixture with a weight ratio of 5:4. Additionally, 0.05 wt% PtTFPP was added into the above mixture of CA and PCL. The mixture was stirred for 3 h before electrospinning. A DC voltage of 18 kV was applied between the syringe tip and an aluminum collector at a distance of 15 cm for electronspinning. The flow rate of the solution was 0.6–1.0 mL/h at room temperature. The obtained CA&PCL film with PtTFPP was then treated in a 0.05 M NaOH aqueous solution for 7 days for deacetylation. The film was rinsed three times with DI water to remove residual NaOH, and after that it was placed in a vacuum oven to dry at room temperature for two days. The obtained film was called F1 (Scheme 1a). 

#### 2.3.2. Preparation of CS Electrospun Nanofibrous Matrix for pH Sensing (F2)

CS-FITC was dissolved in 70% aqueous acetic acid to prepare a 2 wt% solution. A DC voltage of 18 kV was applied. The flow rate of the solution for electrospinning was 1.0 mL/h at humidity below 40%. The obtained films were placed in a dessicator containing a mixed aqueous solution of 2.5% glutaraldehyde (GA) and 10% HCL. After the air inside the dessicator was exhausted, the films were cross-linked for 2 h by the reaction of GA vapor with the amino and/or hydroxyl groups of the CS compositions to increase the stability of the films when used in aqueous solution. Finally, these films (F2, Scheme 1b) were rinsed with DI water three times and dried in a vacuum oven at room temperature.

#### 2.3.3. Preparation of CS Electrospun Nanofibrous Matrix on the Top of F1 for Dual Senor F3

Dual sensor F3 was prepared by electrospinning on the top of F1 film and cross-linked with GA. The procedure was similar to F2, except that the F3 film consists of both the F1 and F2 compositions (Scheme 1c).

#### 2.3.4. Preparation of Nanofibrous CA (F4) or PCL (F5) as Matrices for Comparison with F1

The sensing films using only CA or PCL as the matrix were prepared by electrospinning of 10 wt% CA solution and 15 wt% PCL solution, respectively. Both of the films contained 0.05 wt% PtTFPP. The DC voltage and flow rates of electrospinning are consistent with those of F1.

### 2.4. Characterization of Nanofibrous Matrices

To characterize the fiber morphology, samples were sputter coated with platinum and analyzed with a scanning electron microscope (SEM, MIRA3, TESCAN, Brno, Czech). Contact angle was found with a contact angle tester (AST VCA Optima XE, Beijing, China) to study the hydrophilic and hydrophobic properties of the materials. The phosphorescence microscopy images of microfibrous sensing films were captured with a confocal laser scanning microscope (CLSM, TCS SP8, Leica, Germany).

### 2.5. pH and DO Sensing Performances

#### 2.5.1. Response to pH and DO

The sensing films were cut to dimensions of 1.4 cm × 1.4 cm and placed in a quartz cuvette filled with water or buffer. The fluorescence spectra were excited at 488 nm for pH responses and 405 nm for oxygen sensing and collected by using a JASCO FP-8600 fluorophotometer. Britton–Robison buffers (BR buffers) with various pH values were prepared to evaluate the pH responses of the sensing films. The film was washed at least three times to remove the residual solution when changing buffers. To ensure the accuracy of the experiment, titrations were performed twice to get the average value. DO was adjusted by bubbling mixed gas of N_2_ and O_2_ into the solution in the quartz cuvette for 5 min at a controlled flow rate of 100 SCCM to ensure an equilibrium was reached.

#### 2.5.2. Reversibility of the Sensing Response

Emission intensities at 650 nm from PtTFPP under the excitation wavelength of 405 nm were recorded every 6 min. The measurement switched back and forth between saturated O_2_ and N_2_. Likewise, emission intensities at 520 nm of CS-FITC under the excitation wavelength of 488 nm in acidic (pH = 4.0) or alkaline (pH = 8.0) solutions were measured.

#### 2.5.3. Real-Time Monitoring of the Cell Respiration Process

NIH 3T3 mouse embryonic fibroblast cells were used for cell respiration studies. The cells were cultured with Dulbecco’s modified Eagle’s medium (DMEM) with 10% FBS and 1% antibiotics, and incubated at 37 °C in 5% CO_2_ overnight. The cell density was detected by an automated cell counter (ACC, TC20, Bio-Rad, Hercules, CA, USA), then diluted by fresh medium to appropriate densities (10^6^ cells/mL, 5 × 10^5^ cells/mL, 2.5 × 10^5^ cells/mL and 1.25 × 10^5^ cells/mL) for further measurements. Escherichia coli (ATCC 25922) was cultured by shaking in liquid lysogeny broth (LB) broth at 37 °C overnight. After that, optical density at 600 nm (OD_600 nm_) was measured with a UV–Vis spectrophotometer to determine bacterial density. A value of 1.0 indicated a cell density of 5 × 10^8^ colony-forming units per milliliter (CFU/mL). Then, the bacteria were diluted by LB medium to suitable densities (10^6^ CFU/mL, 5 × 10^5^ CFU/mL, 2.5 × 10^5^ CFU/mL and 1.25 × 10^5^ CFU/mL) for further studies. 

The 200 µL diluted cell and bacterial suspensions with various cell densities were added to a 96-well plate covered with pre-prepared pH/O_2_ responsive membrane F3 at the bottom, and a 70 µL mineral oil seal was used to isolate the cell medium from air to prevent potential oxygen exchange. The wells filled with only nutrient medium without cells and bacteria on the sensing me mbrane were used as a blank control group. The temperature of the microplate reader was set at 37 °C. The excitation wavelengths for pH probes and O_2_ probes were 488 nm and 405 nm, respectively, and the emission intensities at 520 nm and 650 nm were measured with a microplate reader (Cytation 3, BioTek, Winooski, VT, USA) every 5 min. 

### 2.6. In Vitro Biocompatibility Test

#### 2.6.1. MTT Assay

Membrane biocompatibility was evaluated through an MTT assay. Briefly, the disinfected and cleaned films (1 × 4 cm^2^) were immersed in fresh medium for 3 days, then the extraction medium was treated using a 0.20 µm filter. Then, 8 × 10^3^ NIH 3T3 cells per well were seeded in 96-well plates with 100 µL culture medium and incubated for 24 h. Diluted extraction medium (100 µL) of sensing films in a series of concentrations (100, 75, 50, 25 and 0%) was added. The NIH 3T3 cells were treated for 24, 48 and 72 h, then MTT reagent was added. After four hours of incubation, the medium was removed and 150 µL of DMSO was added to dissolve formazan purple crystals. The absorbance of the samples was recorded at 570 nm by a microplate reader.

#### 2.6.2. Cell Proliferation and Spreading

Electrospun sensing films were sterilized by ethanol, then transferred to a 24-well plate. Then, 5 × 10^4^ NIH 3T3 cells per well were seeded with complete medium on the samples. After incubation for 24 h, the samples were rinsed three times with PBS, then fixed with 2.5% GA for one hour. Then, the samples were dehydrated through an alcohol gradient and air dried for 24 h. Dry membranes with cells were sputter coated with platinum to evaluate the cell adhesion on the membranes by SEM. 

## 3. Results and Discussion

### 3.1. Synthesis of CS-FITC Conjugates

**Figure 1:**
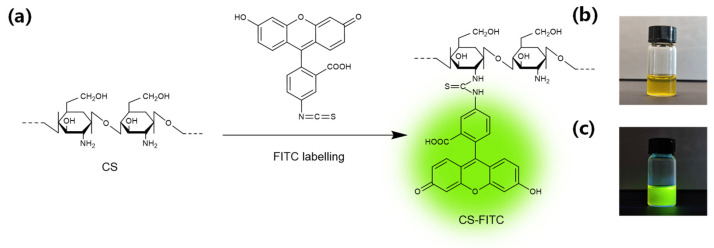
(**a**) Schematic reaction of CS and FITC for the formation of CS-FITC; (**b**) CS-FITC solution in natural light; (**c**) CS-FITC solution under excitation.

The CS-FITC was synthesized by the reaction between the nucleophilic amino group of CS and the isothiocyanate groups of FITC (Figure 1a) [19]. The CS-FITC solution showed a deep yellowish color (Figure 1b), while the solution under excitation showed a green emission (Figure 1c).

### 3.2. Membrane Characterization

**Figure 2:**
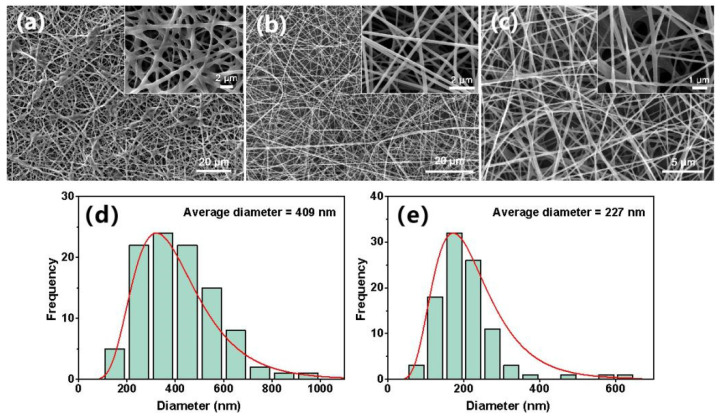
SEM images of F1 (**a**), F2 (**b**) and F3 (**c**) nanofibrous membranes. Diameter distributions of F1 (**d**) and F2 (**e**) nanofibers.

In our study, CA was selected as the supporting matrix for our primary study. In addition, the PCL, which has been proved to be an excellent host material for desired biodegradability properties and satisfactory oxygen permeability [20], was blended with CA material for electrospinning (Scheme 1a). The successful preparation of F1 fiber via electrospinning from DMF solution was evaluated by SEM. F1 was composed of fine nanofibers with an average fiber diameter of 409 ± 157 nm (Figure 2a,d). Additionally, regular, bead-free F2 nanofibers with an average diameter of 227 ± 128 nm (Figure 2b,e) were successfully prepared. Eventually, we obtained a two-layer electrospun membrane F3, and the morphology of F3 is shown in Figure 2c. It can also be seen intuitively in Figure 2c that F2 nanofibers’ average diameter was lower than those of the F1 nanofibers. Other than the surface appearance, Figure S1 shows the SEM images of the cross-section morphologies of F3 film, whereby the two-layer structure can be clearly detected. The thickness of the F1 and F2 layer is about 5 µm and 15 µm, respectively. The increased surface-to-volume ratios are expected to prominently reduce barriers to oxygen diffusion. This structure is vital for applications in reporting localized DO and pH levels in the vicinity of cells, as these nanofibrous structures allow cells to interact closely with the matrix and the incorporated probes [20]. Furthermore, stress–strain curves of F1 membrane were studied. The composite materials of CA and PCL nanofibers showed a high flexibility, the elongation at break was up to 148% (Figure S2). This property can broaden the application of the sensing films in other fields, such as the introduction of indicators on masks.

**Figure 3:**
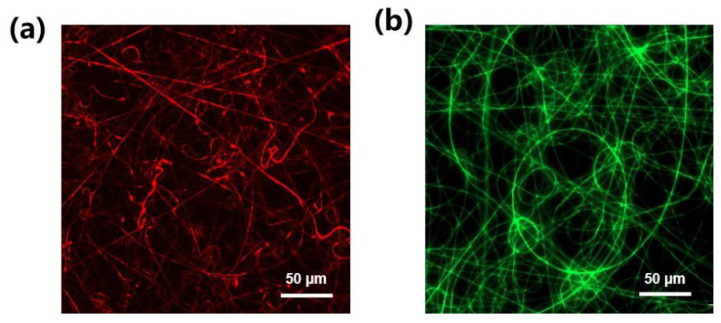
Phosphorescence and fluorescence micrograph of F1 nanofibers (λ_ex_ = 385 nm, λ_em_ = 650 nm) (**a**) and F2 nanofibers (λ_ex_ = 470 nm, λ_em_ = 520 nm) (**b**).

The optical images presented in Scheme 1d show the emissions of three nanofiber membranes. The films give sufficient red and green emissions, respectively, when exposed to ultraviolet light (365 nm). As PtTFPP probes were incorporated in the CA&PCL polymer fibers, red emission from the fibers was further observed under an excitation (Figure 3a). Similarly, since FITC probes were coupled to chitosan, green fluorescence from the fibers was observed (Figure 3b).

**Figure 4:**
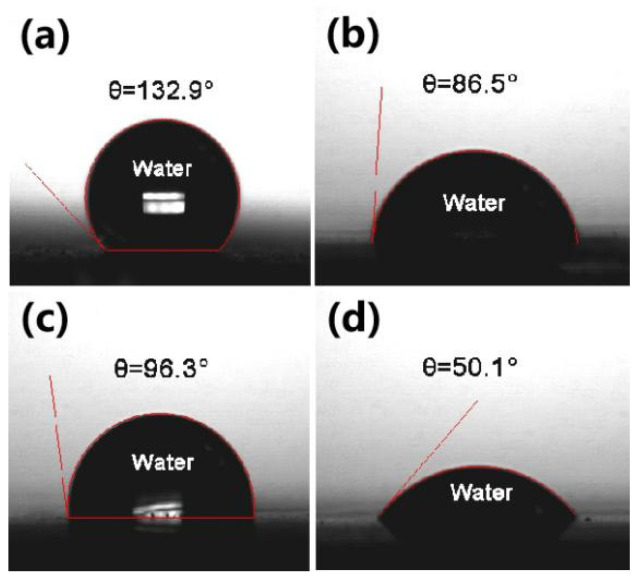
Contact angle of nanofiber membranes, F4 (CA-PtTFPP, (**a**)), F5 (PCL-PtTFPP, (**b**)), F1 (PCL&CA-PtTFPP, (**c**)) and F3 (PCL&CA-PtTFPP/CS-FITC, (**d**)).

The hydrophobicity/hydrophilicity of the four different films were determined by a water contact angle experiment (Figure 4). F4 is the original pure CA electrospun film, and showed strong hydrophobicity with a contact angle of 132.9° (Figure 4a). After blending with PCL, the mixed film F1 was obtained and the angle of the F1 fibers decreased to 96.3° (Figure 4c). The further interaction of CS made the water contact angle of the obtained F3 bilayer nanofibers decrease to 50.1°, showing that the nanofibrous complex surface exhibits hydrophilicity.

### 3.3. Oxygen Sensing Performance

**Figure 5:**
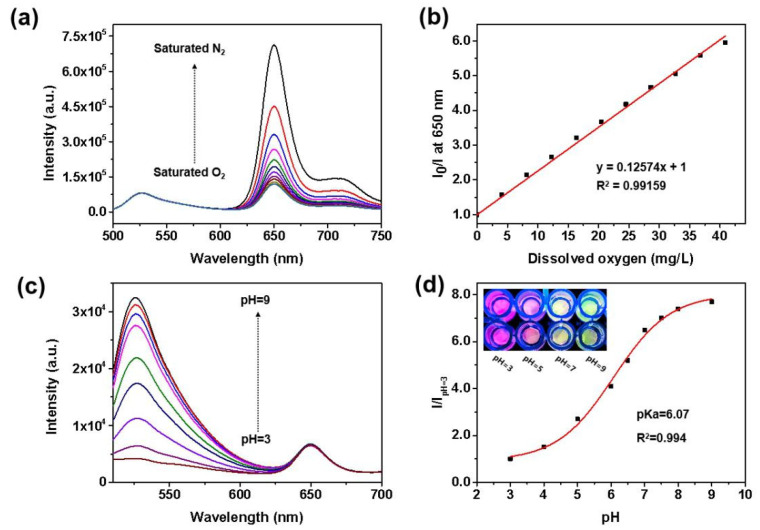
O_2_ and pH responses of CA&PCL-PtTFPP/CS-FITC membrane (F3). Phosphorescent emission spectra of the membrane under different concentrations dissolved oxygen (**a**), Stern–Volmer plots for the F3 membrane (**b**), fluorescence spectra of the F3 membrane in BR buffer at varied pH values (**c**) and plot of pH calibration curve of I/I_pH=3_ versus the pH value (**d**). The inset in (**d**) shows the emission color changes at diverse pHs illuminated by 365 nm LED light.

The sufficient triplet–triplet energy transfer from the oxygen probes (herein, PtTFPP) to oxygen molecules results in the quenching of the emission of the oxygen probes [21]. This kind of quenching behavior is influenced by molecules of probes, the structure of matrix or media and the microenvironment in which the probes are located [21]. The quenching responses of PtTFPP in F3 were tested over a range of DO concentrations. The emission spectra of the film in a series of diverse concentrations of DO are provided in Figure 5a under the excitation of 405 nm. Strong emission at 650 nm of PtTFPP in the absence of O_2_ was observed, and the emission decreased with increasing DO concentrations. The relative intensity change as a function of oxygen concentration follows the Stern–Volmer Equation (1).

(1) $$\frac{I_{0}}{I}=1+K_{SV}\left[O_{2}\right],$$

where *I* is luminescent intensity at 650 nm at different O_2_ concentrations. *I*_0_ denotes a value in the absence of O_2_, *K_SV_* is the Stern–Volmer constant and [O_2_] is the concentration of O_2_.

The results of Figure S3 revealed that the electrospun fiber films as oxygen sensors were more sensitive than the smooth thin film. This is consistent with previous reports, showing that the sensitivity of the electrospun fabricated sensor would be improved due to the much higher surface area-to-volume ratio [22]. Moreover, composite sensing films of CA and PCL were demonstrated to have better sensing performance as compared with the pure CA film (Figure S3). At the excitation wavelength of the PtTFPP (λ = 405 nm), the emission wavelength of FITC at 520 nm had no obvious changes under different DO concentrations, showing that the pH responses were not affected by oxygen.

### 3.4. pH Sensing Performance

Fluorescein has different chemical structures under acidic and basic conditions. The conjugated dianionic tautomer formed in a basic condition showed intense green emission under 488 nm excitation, while the nonconjugated lactone tautomer formed in an acidic condition showed weak or no emission under the 488 nm excitation [23] (Figure S4). The pH response behavior of F3 was studied. As expected, the fluorescence intensity increased with the increase in pH (Figure 5c). The pKa value was determined according to the modified Henderson–Hasselbalch Equation (2).

(2) $$\mathrm{pH}=pK_{a}-log\left[\frac{I_{x}-I_{max}}{I_{min}-I_{x}}\right]$$

where *I_max_* and *I_min_* are the fluorescence intensities of the probe at pH = 9.0 and pH = 3.0, respectively, and *I_x_* is the fluorescence intensity measured at 520 nm under the excitation of 488 nm. 

The pKa for the sensing film was calculated to be 6.07. The dynamic range for pH is from 5.0 to 8.0, indicating its suitableness for biological studies.

The red phosphorescence emitted by the PtTFPP at 650 nm was not influenced by pH. The inset diagram in Figure 5d shows the emission color changes at four different pH values. When the solution environment was basic (pH = 9.0), intense green emission was observed, whereas in acidic solution (pH = 3.0), the green fluorescence disappeared and only the red emission of the oxygen probe was visible. The emission spectrum of FITC and absorption spectrum of PtTFPP are shown in Figure S5. Their spectra overlap, but they are far away from each other in the two-layer film system, failing to reach the standard of a distance of less than 10 nm. Therefore, there is almost no FRET phenomenon between them, which can be ignored. This further shows that red and green channels are less likely to have interference with each other during the simultaneous detection of pH and O_2_, which ensures the effective use of the sensor film when it is used to obtain multiple biological signals in a complex environment.

### 3.5. Reversibility of the Sensing Film F3

**Figure 6:**
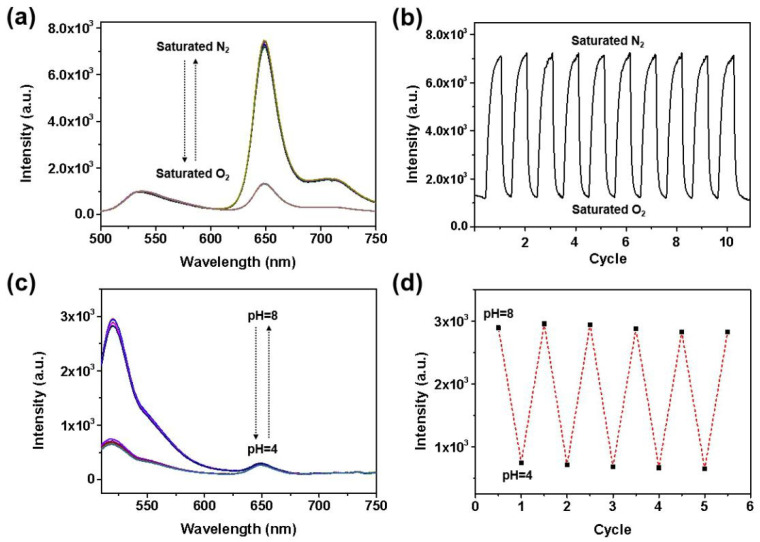
Reversibility of the dual sensing film. Emission spectra (**a**) and reversibility (λ_ex_/λ_em_ = 405 nm/650 nm) (**b**) of the F3 in O_2_-saturated and N_2_-saturated solutions, emission spectra (**c**) and reversibility (λ_ex_/λ_em_ = 488 nm/520 nm) (**d**) of F3 in BR buffer between pH 4.0 and pH 8.0.

The reversibility of the sensing film is vital for real-time biological application. The kinetic phosphorescent spectra of F3 by alternating cycles of saturated O_2_ and saturated N_2_ solution indicated that it exhibits reversible quenching and recovering properties (Figure 6a) and satisfies the demands of a continuous DO monitoring (Figure 6b). Combining synthetic polymers with natural polymers can make their respective advantages complementary to each other. As shown in Figure S6, the optical stability of F5 (pure PCL electrospun sensing films) is not good. Thus, the cooperation between CA and PCL can meet the requirements of improving both the sensitivity (Figure S3) and the stability (Figure 6a,b) of the composite films. The resistance of the sensing film to mechanical deformation was also explored (Figure S7). The sensing performance of the film barely changed after 10 times of folding. This also demonstrated the stability of the film. In Figure 6c,d, the fluorescence intensity between pH 4.0 and 8.0 is also reversible. This detection range can meet the basic needs of biological physiological monitoring. 

### 3.6. Real-Time Monitoring of DO and pH during Cellular Respiration Process

Cells are characterized by vigorous metabolism and diversified metabolic pathways. When oxygen and nutrients are abundant, cells obtain energy mainly through aerobic respiration and the glycolysis pathway [24]. Therefore, the measurement of metabolites such as DO and pH during cellular metabolism can indicate the growth state of cells and bacteria [25]. Here, we used NIH 3T3 cells and *E. coli* as biological models to test the monitoring ability of the sensing film in the process of cell growth. Cells in culture medium with various concentrations were seeded into a 96-well microplate with F3 on the bottom. Mineral oil was used to seal the surface of the medium to avoid the exchange of oxygen in the medium with air. The emissions of oxygen probe and pH probe at 650 nm and 520 nm were recorded during cell growth with the excitation of 405 nm and 488 nm, respectively. 

**Figure 7:**
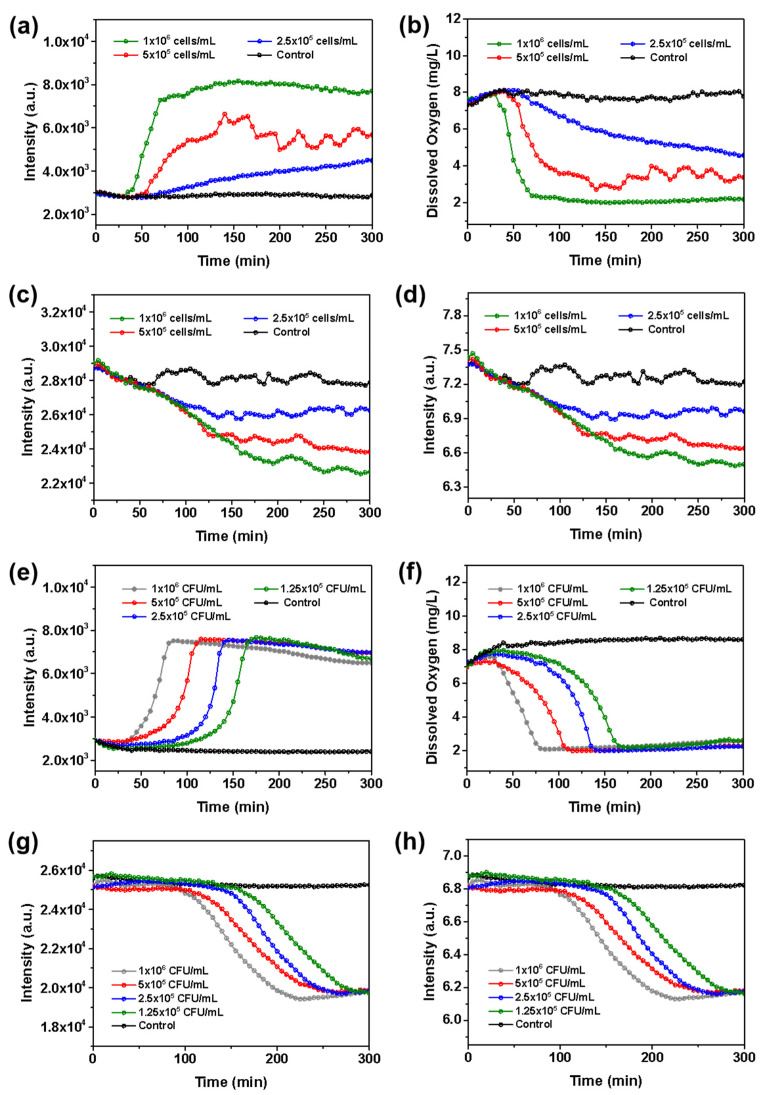
DO and pH monitoring of (**a**–**d**) NIH 3T3 cells and (**e**,**f**) *E. coli.* Luminescence intensity changes of (**a**,**e**) PtTFPP (DO, λ_ex_ = 405 nm, λ_em_ = 650 nm) and (**c**,**g**) CS-FITC (pH, λ_ex_ =488 nm, λ_em_ = 520 nm) during the growth of cells were monitored by F3; DO concentrations (**b**,**f**) and pH values (**d**,**h**) calculated from the calibration curves.

As illustrated in Figure 7a,e, the emission intensities of oxygen probes gradually increased with the prolongation of incubation time. The corresponding DO concentrations were calculated and are given in Figure 7b,f. This phenomenon can be mainly attributed to the depletion of oxygen in cellular respiration. Aerobic respiration is the main way for aerobic organisms to obtain energy through the complete oxidation of matrix. It is a biological oxidation process in which molecular oxygen acts as the final electron acceptor, and thus oxygen is consumed in this process (Figure S8). In addition, high bacterial densities were accompanied by high rates of fluorescence change, due to rapid consumption of oxygen by massive bacterial respiration [26].

The acidification of the medium via the catabolism of carbohydrate is proved by the fluorescence changes of FITC, summarized in Figure 7c,d,g,h. With the increase in cell concentration and the prolonging of incubation time, the emission density at 520 nm decreased, indicating that the medium showed a tendency of acidification. Carbohydrates in the culture medium are consumed by cells and bacteria as carbon sources [24], and electrons supplied during metabolism are transferred to the membrane electron transport chain, where a series of redox reactions release protons, leading to acidification of the growth environment (Figure S8). 

Bacteria proliferate by a simple binary fission, and thus have an astonishing rate of proliferation and metabolism compared to cells [27]. The oxygen consumption and extracellular acidification manners of cells and bacteria are similar, when they are in an ultrahigh density (Figure 7a,e). However, as the density dropped, the differences between them became more and more pronounced. It seems that the cells need more time than bacteria to reach DO and pH equilibrium, revealing that the cells metabolize respiration at a relatively slow rate.

Some interference was also found in Figure 7a,e. The phosphorescence intensities of the O_2_ probes decreased slightly during the experiment. This phenomenon is mainly attributed to the temperature instability of mixtures, which affected the oxygen probe of PtTFPP slightly [28]. Another interesting finding is that the variation trend of the pH was much slower than that of oxygen, which was based on the fact that the buffering capability of the culture medium retards the pH variations in the environment. Therefore, the F3 displayed the ability for simultaneously monitoring the DO and pH changes in microbial environments. 

### 3.7. In Vitro Biocompatibility Test

**Figure 8:**
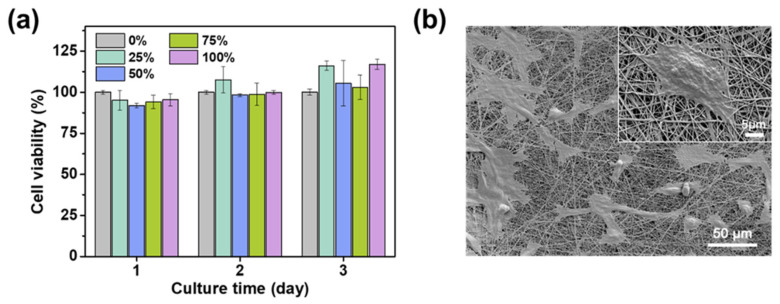
(**a**) Cytotoxity of electrospinning membrane extract at different concentrations after 1, 2, 3 days of incubation; (**b**) SEM morphology of NIH 3T3 cells seeded on the surfaces of F3 membrane after 24 h of incubation.

The MTT assay indicated that more than 90% of the NIH 3T3 cells were viable compared with the control group (Figure 8a), which also means all the membranes’ extracts had no cytotoxicity. The above results were further confirmed, and the morphology of the cell attachment and spreading on the membranes after 24 h of culture were seen from the SEM images (Figure 8b). Most cells on the F3 membranes were intensively adhered to the surfaces and showed spindle-like shapes. These characteristics revealed the good biocompatibility of F3 nanofibrous membranes for NIH 3T3 proliferation. All of the above results indicate the outstanding potential of F3 membranes for biological application.

The growth curve of *E. coli* was measured by the OD_600 nm_ approach. Growth was characterized by a rapid initial exponential growth phase, and reached the platform stage in less than 3 h (Figure S9). There was no significant difference in the growth curve between the experimental group with the sensing films and the control group, indicating the low toxicity of the material. 

## 4. Conclusions

A thin film sensor based on composite materials has been successfully prepared by an electrospinning strategy. The film can be used to sense and image both pH and DO simultaneously without interference from each other’s signals, which has the potential for biological applications. The unique chemical stability of the film ensures its suitability in biological environments. The sensing film was successfully applied to record the change in extracellular DO and pH during the process of cell growth, which can help understand the metabolism behavior of cells. Compared to sensors based on a single function, this multifunctional sensor design strategy is also expected to integrate simultaneous luminous imaging of multiple biological signals. These specific properties mean the sensing film is promising to achieve in situ dynamic monitoring in many fields such as environmental monitoring, biological manufacturing, wound care, etc., with great research and application value.

## Figures and Tables

**Table 1:** Compositions of electrospun sensing films of F1–F5.

Films	Compositions	Probe Molecules	Response	Cross-Linking
F1	CA:PCL = 5:4 (weight ratio)	PtTFPP	DO	-
F2	CS	FITC	pH	GA vapor
F3	F1 + F2	PtTFPP and FITC	DO and pH	GA vapor
F4	CA	PtTFPP	DO	-
F5	PCL	PtTFPP	DO	-

## Data Availability

The data presented in this study are contained within the article and are also available in the Supplementary Materials.

## Supplementary Materials

The following are available online: Figure S1: SEM images of the film cross-section displaying the thickness of the two-layer membrane. Figure S2: The stress–strain behavior of F1 film. Figure S3: Stern–Volmer plots for the pure CA and CA&PCL in the form of electrospun films or smooth thin films. Figure S4: Structural changes in the fluorescein moieties in strong basic and acidic conditions. Figure S5: The absorption spectra of PtTFPP and emission spectra of FITC. Figure S6: Reversibility of the PCL electrospun film in O_2_-saturated and N_2_-saturated solutions. Figure S7: Response of the F1 sensing films before and after 10 times of folding. Figure S8: Scheme of aerobic respiration and glycolysis. Figure S9: OD_600 nm_ measurement of *E. coli* with and without CS film in neutral medium (pH = 7.0).

## Sample Availability

Samples of the compounds F1–F5 are available from the authors.
