# Supplementary material for: A Dual pH/O2 Sensing Film Based on Functionalized Electrospun Nanofibers for Real-Time Monitoring of Cellular Metabolism
Source: Molecules. 2022 Feb 28;27(5):1586. doi: 10.3390/molecules27051586 (PMC8911770; doi:10.3390/molecules27051586)
Supplement: Supplementary file 1 [file molecules-27-01586-s001.zip › molecules-1606075-supplementary.pdf]

Supplementary information

A dual pH/O_2 sensing film based on functionalized electrospun nanofibers for real-time monitoring of cellular metabolism

Dongyan Zhou, Hongtian Liu, Juewei Ning, Ge Cao, He Zhang, Mengyu Deng*, Yanqing Tian*

Department of Materials Science and Engineering, Southern University of Science and Technology, No 1088 Xueyuan Blvd, Xili, Nanshan District, Shenzhen, Guangdong, 518055, China,

*Corresponding author**

E-mail: tianyq@sustech.edu.cn, ORCID: http://orcid.org/0000-0002-1441-2431

Contents

Figure S1: SEM images of the film cross-section displaying the thickness of the two-layer membrane. 2

Figure S2: The stress-strain behavior of F1 film. 2

Figure S3: Stern-Volmer plots for the pure CA and CA&PCL in the form of electrospun films or smooth thin films. 3

Figure. S4: Structural changes of the fluorescein moieties at strong basic and acidic conditions. 3

Figure. S5: The absorption spectra of PtTFPP and emission spectra of FITC. 4

Figure S6: Reversibility of the PCL electrospun film in O_2-saturated and N_2-saturated solutions. 4

Figure S7: Response of the F1 sensing films before and after 10 times of folding. 5

Figure. S8: Scheme of aerobic respiration and glycolysis. 5

Figure S9: $OD_{600\ nm}$ measurement of *E. coli* with and without CS film in neutral medium (pH=7.0). 6

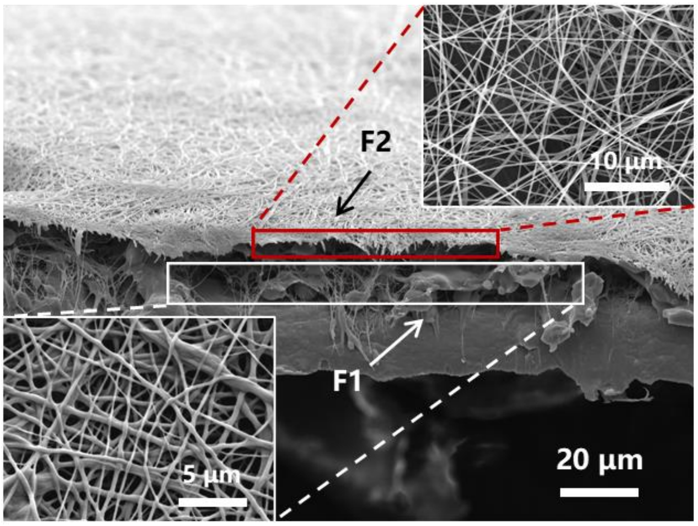

Figure S1: SEM images of the film cross-section displaying the thickness of the two-layer membrane.

The part marked in white is the F1 layer and the part marked in red is F2 layer. Underneath the two layer of membranes is the aluminum foil.

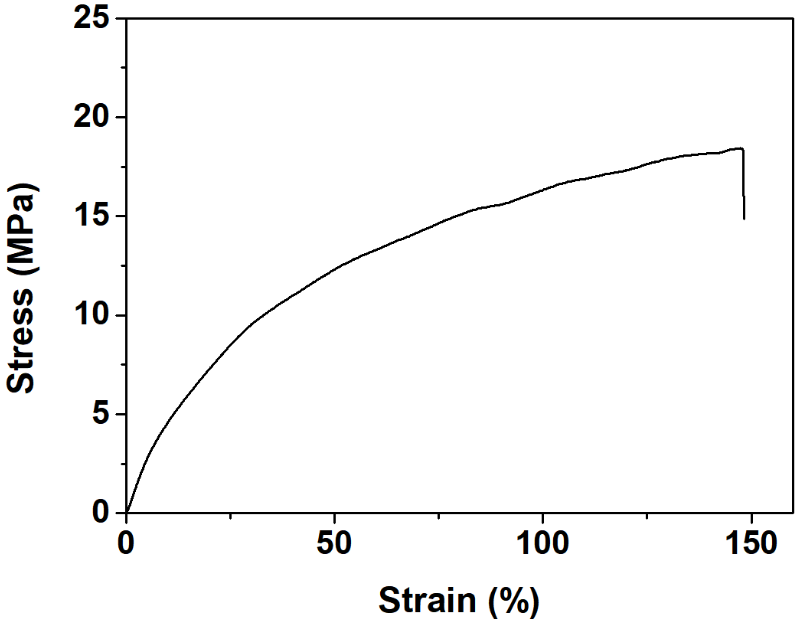

Figure S2: The stress-strain behavior of F1 film.

The nanofibrous membranes were evaluated for a stress-strain response using a tensile tester (Instron 5566, Illinois Tool Works Inc., USA).

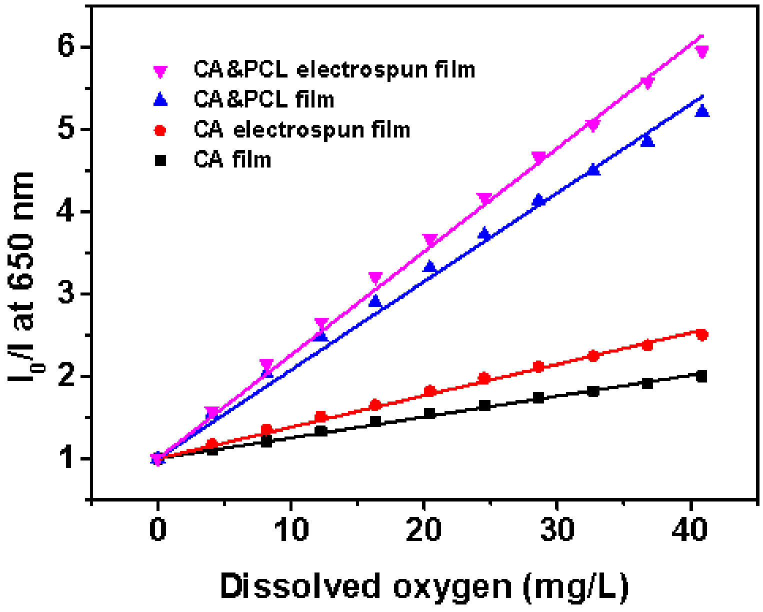

Figure S3: Stern-Volmer plots for the pure CA and CA&PCL in the form of electrospun films or smooth thin films.

The smooth film of CA and CA&PCL were prepared by adding the matrix solution onto the surface of quartz glass and covered with a clean cover slip to make a sandwich structure. The thickness is consistent with that of the electrospun films. The smooth films were obtained after the solvent evaporated.

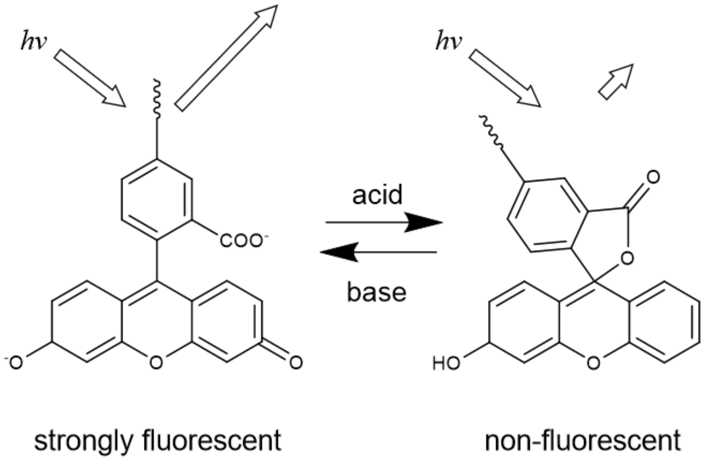

Figure. S4: Structural changes of the fluorescein moieties at strong basic and acidic conditions.

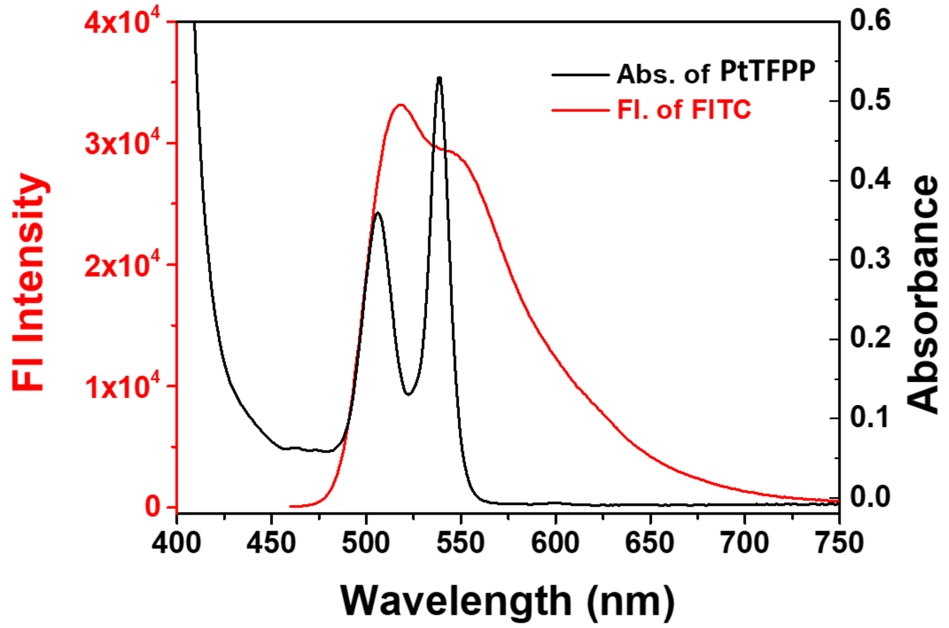

Figure. S5: The absorption spectra of PtTFPP and emission spectra of FITC.

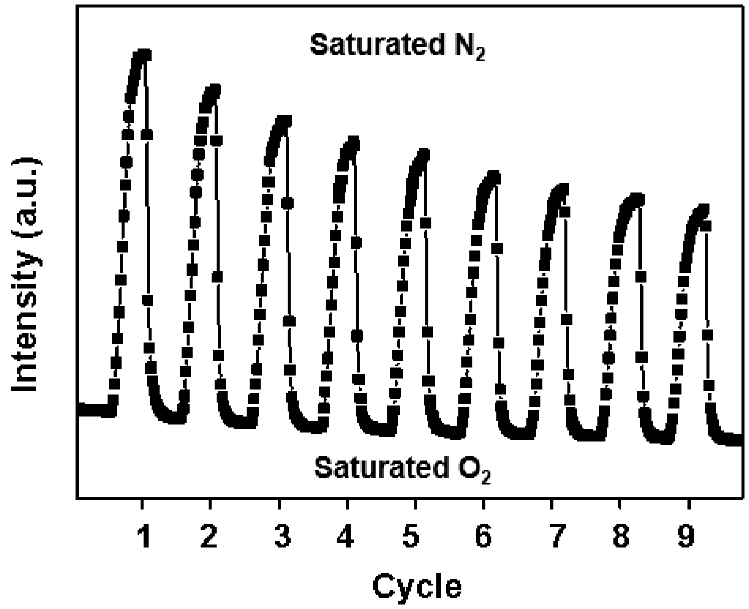

Figure S6: Reversibility of the PCL electrospun film in O_2-saturated and N_2-saturated solutions.

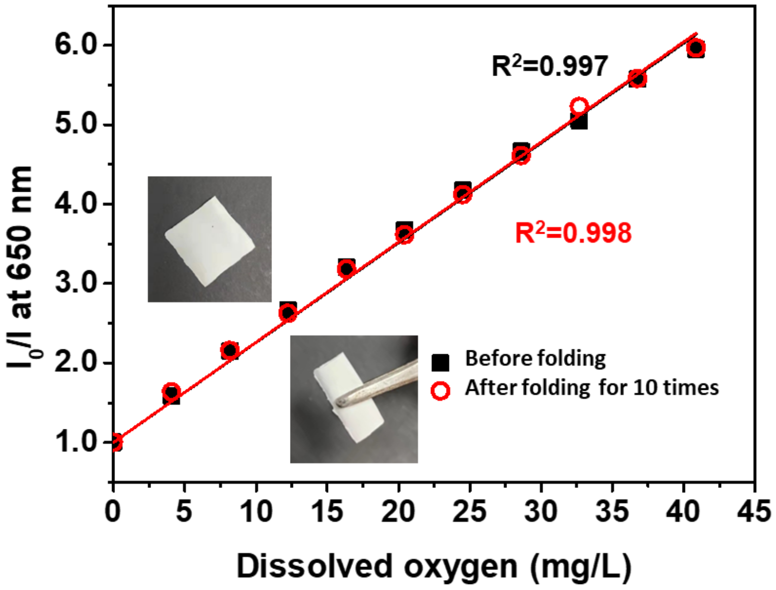

Figure S7: Response of the F1 sensing films before and after 10 times of folding.

After 10 cycles of folding the film with a tweezer, the DO response was measured.

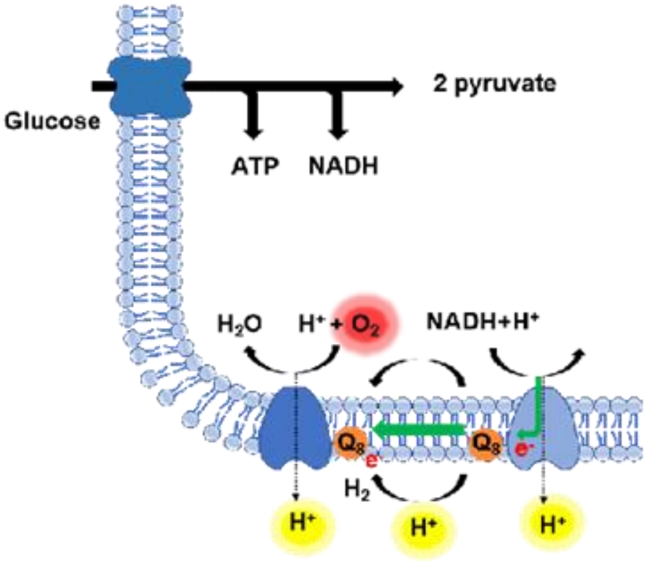

Figure. S8: Scheme of aerobic respiration and glycolysis.

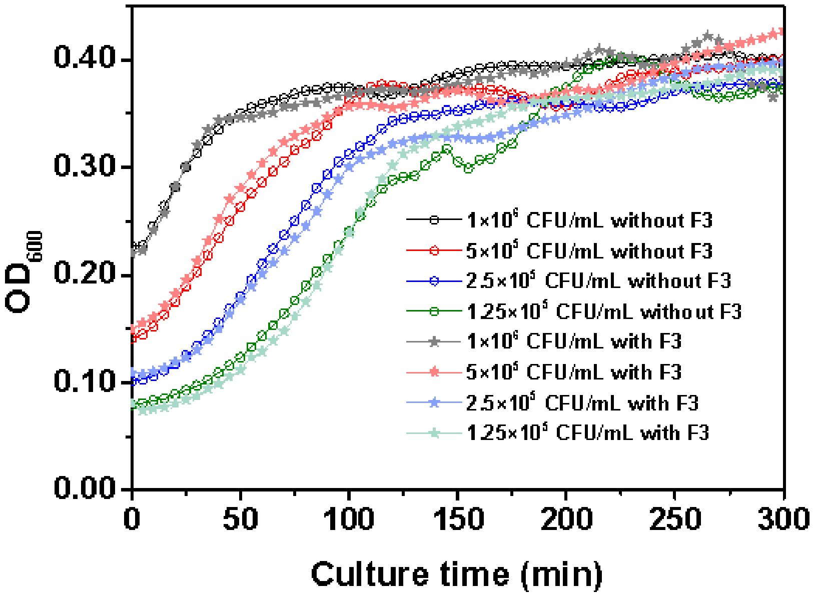

Figure S9: $OD_{600\ nm}$ measurement of *E. coli* with and without CS film in neutral medium (pH=7.0).
